# Oncostatin M is overexpressed in NASH‐related hepatocellular carcinoma and promotes cancer cell invasiveness and angiogenesis

**DOI:** 10.1002/path.5871

**Published:** 2022-03-07

**Authors:** Giovanni Di Maira, Beatrice Foglia, Lucia Napione, Cristian Turato, Marina Maggiora, Salvatore Sutti, Erica Novo, Maria Alvaro, Riccardo Autelli, Sebastiano Colombatto, Federico Bussolino, Patrizia Carucci, Silvia Gaia, Chiara Rosso, Alessandra Biasiolo, Patrizia Pontisso, Elisabetta Bugianesi, Emanuele Albano, Fabio Marra, Maurizio Parola, Stefania Cannito

**Affiliations:** ^1^ Department of Clinical and Experimental Medicine and Center Denothe University of Firenze Firenze Italy; ^2^ Department of Clinical and Biological Sciences, Unit of Experimental Medicine and Clinical Pathology University of Torino Torino Italy; ^3^ Laboratory of Vascular Oncology Candiolo Cancer Institute – FPO IRCCS (Istituto di Ricovero e Cura a Carattere Scientifico) Torino Italy; ^4^ Department of Applied Science and Technology Politecnico di Torino Torino Italy; ^5^ Department of Molecular Medicine University of Pavia Pavia Italy; ^6^ Department of Health Sciences and Interdisciplinary Research Center for Autoimmune Diseases University Amedeo Avogadro of East Piedmont Novara Italy; ^7^ Department of Oncology University of Torino Torino Italy; ^8^ Division of Gastroenterology Città della Salute e della Scienza University‐Hospital Turin Italy; ^9^ Department of Medical Sciences University of Torino Torino Italy; ^10^ Department of Medicine University of Padova Padova Italy

**Keywords:** oncostatin M, NAFLD, NASH, EMT, hepatocellular carcinoma, invasiveness, angiogenesis, metastasis

## Abstract

Oncostatin M (OSM) is a pleiotropic cytokine of the interleukin (IL)‐6 family that contributes to the progression of chronic liver disease. Here we investigated the role of OSM in the development and progression of hepatocellular carcinoma (HCC) in non‐alcoholic fatty liver disease (NAFLD)/non‐alcoholic steatohepatitis (NASH). The role of OSM was investigated in (1) selected cohorts of NAFLD/NASH HCC patients, (2) liver cancer cells exposed to human recombinant OSM or stably transfected to overexpress human OSM, (3) murine HCC xenografts, and (4) a murine NASH‐related model of hepatic carcinogenesis. OSM was found to be selectively overexpressed in HCC cells of NAFLD/NASH patients, depending on tumor grade. OSM serum levels, barely detectable in patients with simple steatosis or NASH, were increased in patients with cirrhosis and more evident in those carrying HCC. In this latter group, OSM serum levels were significantly higher in the subjects with intermediate/advanced HCCs and correlated with poor survival. Cell culture experiments indicated that OSM upregulation in hepatic cancer cells contributes to HCC progression by inducing epithelial‐to‐mesenchymal transition and increased invasiveness of cancer cells as well as by inducing angiogenesis, which is of critical relevance. In murine xenografts, OSM overexpression was associated with slower tumor growth but an increased rate of lung metastases. Overexpression of OSM and its positive correlation with the angiogenic switch were also confirmed in a murine model of NAFLD/NASH‐related hepatocarcinogenesis. Consistent with this, analysis of liver specimens from human NASH‐related HCCs with vascular invasion showed that OSM was expressed by liver cancer cells invading hepatic vessels. In conclusion, OSM upregulation appears to be a specific feature of HCC arising on a NAFLD/NASH background, and it correlates with clinical parameters and disease outcome. Our data highlight a novel pro‐carcinogenic contribution for OSM in NAFLD/NASH, suggesting a role of this factor as a prognostic marker and a putative potential target for therapy. © 2022 The Authors. *The Journal of Pathology* published by John Wiley & Sons Ltd on behalf of The Pathological Society of Great Britain and Ireland.

## Introduction

Non‐alcoholic fatty liver disease (NAFLD) is emerging as the most common cause of chronic liver disease (CLD) worldwide, being characterized by a 25% global prevalence in the general population and an even higher prevalence among patients with obesity and/or type II diabetes [1, 2, 3, 4, 5, 6, 7]. According to current estimates, up to 30% of individuals in the general population of the USA and Europe have NAFLD [1]. NAFLD is a spectrum of conditions that mainly include simple steatosis, but a significant number of patients also develop non‐alcoholic steatohepatitis (NASH). NASH represents the progressive form of disease characterized by hepatocyte injury and lobular inflammation, possibly progressing towards advanced fibrosis, cirrhosis, and, eventually, liver failure [1, 4, 5, 6, 7]. NAFLD is also emerging as a major cause of hepatocellular carcinoma (HCC), the most common primary liver cancer, currently representing the third leading cause of cancer mortality worldwide [8, 9, 10]. HCC carries a high mortality burden, due to still unsatisfying therapeutic options. Furthermore, differently from what is reported for CLD due to other etiologies, NASH‐related HCC is increasingly diagnosed in non‐cirrhotic patients [8, 9, 10]. HCC development and progression is characterized by multi‐step accumulation of genetic and epigenetic alterations, resulting in HCC heterogeneity in terms of phenotype and functional signatures [11], making it difficult to unveil disease mechanisms as well as early diagnosis and the choice of the best therapeutic strategies.

Changes associated with chronic liver diseases (CLDs), including fibrogenesis, angiogenesis, and inflammation, play a critical role in HCC, emphasizing the relevance of tumor–stroma interactions [12, 13]. Oncostatin M (OSM) is a pleiotropic and multifunctional cytokine belonging to the IL‐6 family, produced by different cell types, including activated monocytes/macrophages, T cells, dendritic cells, and neutrophils [14]. OSM acts on a wide variety of cells engaging two distinct heterodimeric receptor complexes, gp130/OSM receptor beta (OSMRβ) and gp130/leukemia inhibitory factor receptor beta (LIFRβ). OSMRβ mediates the majority of OSM effects [14] by activating the Janus kinase/signal transducer and activator of transcription (JAK/STAT) pathway [14], as well as the Ras–MAPK, ERK1/2, and p38 pathways [15, 16].

OSM has been shown to participate in tumor progression by favoring proliferation, cell growth, and neo‐angiogenesis, as well as by inducing epithelial–mesenchymal transition (EMT) in breast cancer cell lines [6, 17, 18, 19, 20]. The expression of OSM and its receptors is increased in the livers of cirrhotic patients [21], and OSM has been shown to orchestrate hypoxia‐modulated processes, including regeneration, fibrogenesis, and angiogenesis [13, 22, 23, 24]. OSM serum levels are also elevated in cirrhotic patients both in the presence and in the absence of HCC [13], suggesting its possible contribution in CLD progression and HCC development [25]. However, the actual involvement of OSM in the mechanisms of HCC progression is still under‐investigated. Here, we found that OSM is overexpressed in HCC arising on a NAFLD/NASH background and correlates with disease outcomes. In cells and murine models, OSM contributes to HCC progression, switching on EMT and promoting angiogenesis, thus favoring metastatic dissemination.

## Materials and methods

Laboratory reagents used in the present study and not mentioned in the following sections are listed in Supplementary materials and methods.

### Cells and culture conditions

HepG2 cells (ATCC‐HB‐8065 from ATCC, Manassas, VA, USA) were stably transfected with a human oncostatin M cDNA in order to overexpress OSM (H/OSM) or with the empty vector (pCMV6) alone (H/V6) and selected with G418 disulfate salt solution (Merck‐Sigma Aldrich, St Louis, MO, USA) as described previously [26]. HepG2 naïve cells and HepG2 transgenic cells maintained in Dulbecco's modified Eagle's medium low glucose (DMEM, 1000 mg/l, from Merck‐Sigma Aldrich) were seeded in normoxic conditions to obtain the desired sub‐confluence level (65–70%) and for performing the subsequent experimental analyses.

Human umbilical vein endothelial cells (HUVECs) were isolated, maintained, and used as pools of five different donors to minimize cell variability as previously described [27]. Collection of umbilical cords for isolation of HUVECs was under an agreement between the University of Torino and the Azienda Ospedaliera Ordine Mauriziano di Torino Hospital, protocol number 1431 02/09/2014. Informed consent was obtained from all subjects involved.

### Human samples

For this study, we analyzed different cohorts of patients: (1) a selected cohort of NAFLD/NASH patients at different stages of disease progression (NAFLD patients with fatty liver only, *n* = 6; NASH patients, either fibrotic or not fibrotic, *n* = 15; NASH‐related cirrhotics, *n* = 32; NASH‐related HCC patients, *n* = 30) referred to the Division of Gastro‐Hepatology of the University of Turin; (2) liver specimens from patients with cirrhosis (*n* = 50) and patients with cirrhosis carrying HCC (*n* = 54), with similar mixed etiology (alcohol, HCV, HBV, autoimmune, metabolic), referred to the Internal Medicine and Hepatology Clinic, Department of Medicine, University of Padova. Blood samples were collected at the time of outpatient visit, while tissue samples were collected at the time of liver tumor resection or liver transplantation. All subjects gave informed consent to the analysis, and the study protocols, which conformed to the ethical guidelines of the 1975 Declaration of Helsinki, were approved by the ethics committees of the Azienda Ospedaliera Universitaria Città della Salute (n.0125391, 18 December 2018, Practice N. CS2/880), Torino, Italy and the Azienda Ospedaliera‐Università, Padova, Italy (11 December 2006), respectively. The clinical and biochemical features of the NAFLD/NASH patients and the HCC patients are reported in supplementary material, Tables S1–S3.

### Animal experiments

These experiments complied with EU and national ethical guidelines for animal experimentation, and experimental protocols were approved by the Animal Ethics Committee of the University of Oriental Piedmont, Novara, Italy, and the Italian Ministry of Health (authorization No 1114/2016). In this study, wild‐type C57BL/6 mice (Harlan Laboratories, Indianapolis, IN, USA) were submitted to (a) an established liver carcinogenic protocol involving single administration of diethylnitrosamine (DEN, 25 mg/kg BW, i.p., Merck‐Sigma Aldrich) at the age of 2 weeks to mice, which were then fed from the age of 6 weeks on a choline‐deficient l‐amino acid‐defined (CDAA) diet (Laboratorio Dottori Piccioni, Gessate, Italy) for an additional 26 weeks [28] or (b) a CDAA diet only or (c) a choline‐sufficient l‐amino acid‐defined (CSSA) diet (Laboratorio Dottori Piccioni) for 24 weeks starting from the age of 6 weeks as previously described [29]. Mice were kept under specific pathogen‐free conditions and maintained with free access to pellet food and water. Liver samples were obtained and immediately used/processed for morphological or molecular biology analyses or frozen and thereafter maintained at −80 °C for further analysis.

### Murine xenograft model

H/V6 and H/OSM cells (1.5 × 10^6^ cells) were resuspended in DMEM‐low glucose with Matrigel at a 1:1 ratio. The cell suspension was injected subcutaneously in a total volume of 0.1 ml into the right flanks of 6‐week‐old female athymic nude (nu/nu) mice (Envigo, Prattville, AL, USA). Ten mice were injected with control cells [HepG2 cells transfected with the empty vector (pCMV6), H/V6, from OriGene, Rockville, MD, USA] and ten mice with cells [HepG2 cells transfected with Oncostatin M (OSM) Human Myc‐DDK‐tagged ORF Clone, H/OSM, from OriGene]. Animal health was monitored daily and if a palpable tumor appeared, its size was measured every 2–3 days with a caliper. Tumor volumes were calculated using the formula *V* = 4/3π (1/2 length × 1/2 width × 1/2 depth). All animals received humane care according to the criteria outlined in the Guide for the Care and Use of Laboratory Animals prepared by the National Academy of Sciences and published by the National Institutes of Health (NIH publication 86‐23, revised 1985) [30], and experiments were performed after obtaining permission of local authorities. These experiments complied with EU and national ethical guidelines for animal experimentation, and experimental protocols were approved by the Animal Ethics Committee of the University of Florence, Florence, Italy, and the Italian Ministry of Health (authorization No 170‐2018‐PR).

### Matrigel invasion assay and analysis of metalloproteinase activity

Cell invasion assays were performed by employing Boyden chambers equipped with 8‐μm pore size polyvinylpyrrolidone‐free polycarbonate filters that were coated with 50 μg/ml Matrigel solution (BD Biosciences, Franklin Lakes, NJ, USA); analyses of MMP‐2 were evaluated as described previously in detail [31]. Invasiveness was evaluated by counting crystal violet‐stained cells that invaded the Matrigel and migrated to the lower surface of Matrigel‐coated polycarbonate filters, using a Zeiss microscope (Zeiss, Oberkochen, Germany) equipped with bright‐field optics. For each filter/Matrigel, the number of cells in ten randomly chosen fields was counted, and the counts were averaged (means ± SD). Analysis of the activity of MMP‐2 was performed on culture medium by means of gelatin zymography, as detailed previously [31]. Because FBS contains MMPs, cells were cultured in 1% serum DMEM‐low glucose. Afterwards, the supernatant was collected, supplemented with Laemmli sample buffer, and subjected to 10% SDS‐PAGE with 1 mg/ml gelatin under non‐denaturing and non‐reducing conditions.

### Protein extraction procedures, western blotting, and analysis

The procedures for protein extraction are detailed in Supplementary materials and methods.

Lysates or extracts were then subjected to sodium dodecyl sulfate‐polyacrylamide gel electrophoresis on 13.5%, 10%, or 7.5% acrylamide gels. After running, the gels were placed onto a nitrocellulose membrane and an electrical current was applied, inducing the proteins to migrate from the gel onto the membrane. Transfer of proteins to the membrane was checked using Ponceau S staining before the blocking step. The blots were then incubated with the desired primary antibodies, then with peroxidase‐conjugated anti‐mouse or anti‐rabbit immunoglobulins in Tris‐buffered saline–Tween containing 2% (w/v) non‐fat dry milk, and finally developed with the ECL reagents following the manufacturer's instructions (Amersham Pharmacia Biotech Inc, Piscataway, NJ, USA). Sample loading was evaluated by reblotting the same membrane with the unphosphorylated form of protein or with β‐actin as well as α‐tubulin antibodies (from Merck‐Sigma Aldrich). Analysis of western blots was performed as previously described [31].

### Spheroid‐based sprouting angiogenesis *in vitro* assay

HUVECs were suspended at a density of 4000 cells/ml in culture medium containing 20% Methocel stock solution (12 mg/ml carboxymethyl cellulose in M199, Merck‐Sigma Aldrich) and 20% FCS, as described previously [32, 33]. Additional details are provided in Supplementary materials and methods.

### RT‐qPCR

RNA extraction, complementary DNA synthesis, and quantitative real‐time PCR (qPCR) reactions were performed on cell samples as well as on human or mouse specimens as described previously [34, 35]. Levels of mRNA were measured by RT‐qPCR, using the SYBR® Green method as previously described [29]. The sequences of the oligonucleotide sequence of primers used for RT‐qPCR are provided in supplementary material, Table S4.

### Immunohistochemistry and hematoxylin and eosin staining

Paraffin‐embedded tumor specimens from the xenograft experiments study were immunostained as described previously [29, 36]. Details of the antibodies used are provided in Supplementary materials and methods. In brief, paraffin sections (2 μm thick) on poly‐l‐lysine‐coated slides were incubated with an anti‐DDK (FLAG) monoclonal antibody (dilution 1:1000, OriGene). After blocking endogenous peroxidase activity with 3% v/v of 30% hydrogen peroxide stock solution (Sigma Merck‐Aldrich) and performing microwave antigen retrieval in sodium citrate buffer (pH 6), primary antibodies were detected using the EnVision, HRP‐labeled System (DAKO, from Agilent, Santa Clara, CA, USA) and visualized using 3,3'‐diaminobenzidine as substrate. For negative controls, the primary antibodies were replaced by isotype‐ and concentration‐matched irrelevant antibodies. Conventional histological staining (H&E, Agilent) was performed on paraffin sections (2 μm thick) in order to evaluate the overall vascularization of tumor tissues.

### Quantification of OSM and VEGF

Human sera from (1) NAFLD/NASH‐related cirrhotic and HCC patients, (2) cirrhotic and HCC patients from mixed etiology, and (3) patients with an early stage of liver disease (steatosis and fibrogenic NASH) were processed in order to evaluate OSM serum levels by employing a Human Oncostatin M (OSM) ELISA kit (EHOSM; Invitrogen, Thermo Fisher Scientific, Waltham, MA, USA) according to the manufacturer's instructions. In addition, conditioned medium collected from H/pCMV6 or H/OSM cells was analyzed to quantify VEGF release into the culture medium by using Human VEGF‐A Platinum ELISA (BMS277/2; Invitrogen, Thermo Fisher Scientific) according to the manufacturer's instructions.

### Statistical analyses

Statistical analyses were performed using GraphPad Prism 6.01 statistical software (GraphPad Software Inc, San Diego, CA, USA). Student's *t*‐test or one‐way ANOVA test with Tukey's correction for multiple comparisons as well as Mann–Whitney non‐parametric tests and unpaired *t*‐tests with Welch's correction were employed to evaluate the statistical significance of the data, with *p* < 0.05 considered as significant. Kolmogorov–Smirnov tests were used for preliminary assessment of the normality of distribution. *In vitro* data are presented as bar charts with means ± SEM and were obtained from average measures of at least three independent experiments.

## Results

### Oncostatin M is upregulated in NAFLD/NASH patients carrying HCC

**Figure 1:**
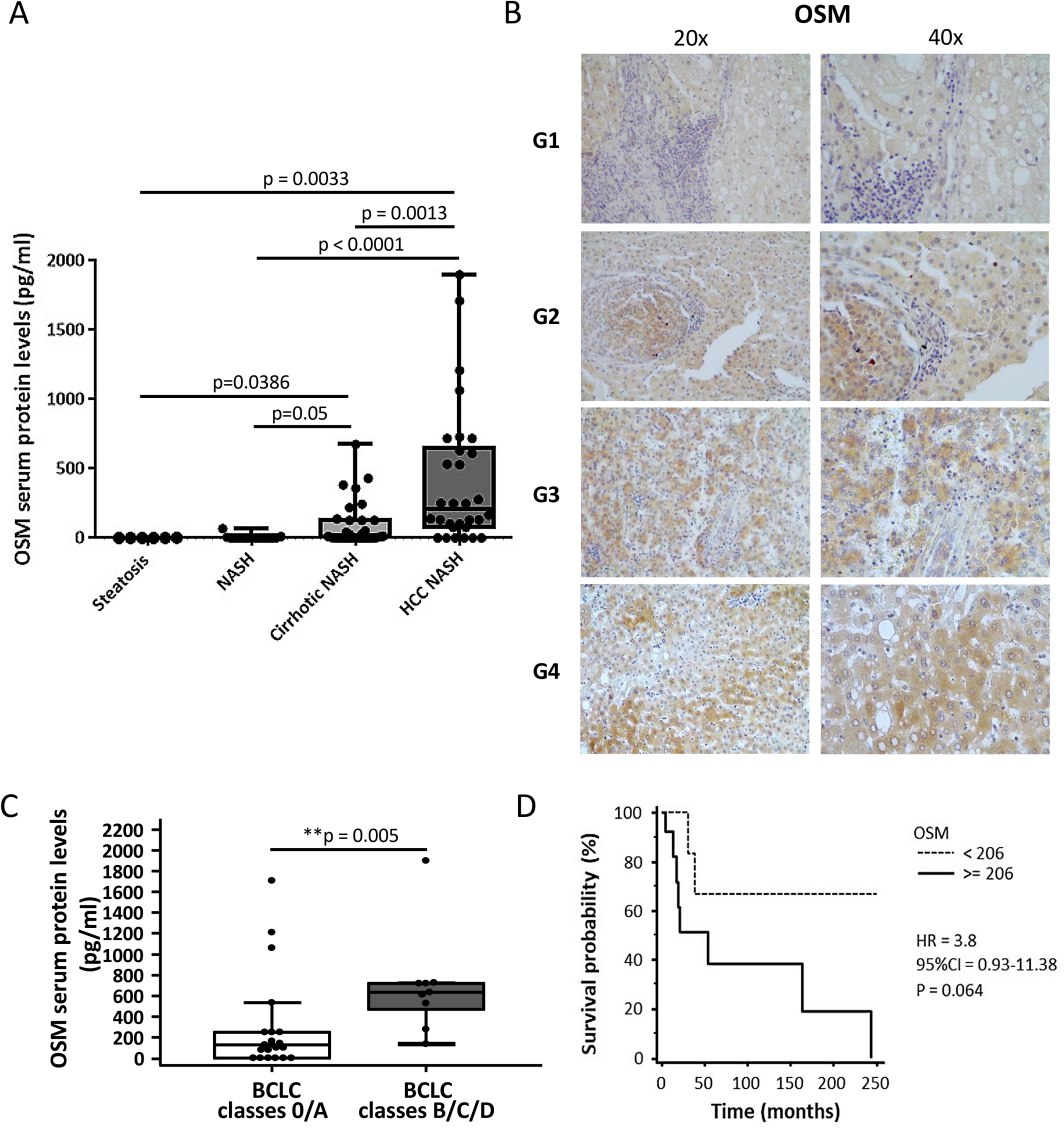
OSM is specifically associated with the development of NAFLD/NASH‐associated HCC. (A) Human OSM serum levels in relation to different stages of the liver disease. One‐way ANOVA test with Tukey's correction for multiple comparisons (*p < 0.05, **p < 0.01, ***p < 0.001). Boxes include the values within the 25th and 75th percentiles, and the horizontal bars present the medians. The extremities of the vertical bars comprise the minimum and the maximum value. (B) IHC analysis for OSM performed on paraffin‐embedded sections of NAFLD/NASH‐related HCC specimens from G1 to G4 tumor grading. Original magnifications are indicated. (C) OSM serum levels of NASH‐related HCC patients according to the Barcelona Clinic Liver Cancer (BCLC) staging system by combining grade 0/A and grades B/C/D. Data are presented as median and 95% CI of the median (**p < 0.01). (D) Kaplan–Meier curves of survival according to OSM protein levels. Statistical analysis was performed using a log‐rank (Mantel–Cox) test.

We first analyzed OSM serum levels and tissue expression in a cohort of 83 NAFLD/NASH patients stratified according to progression of the disease (Figure 1A and supplementary material, Tables S1 and S2). OSM was almost undetectable in the sera of patients with simple steatosis or steatohepatitis but significantly higher in those with cirrhosis. Remarkably, OSM levels were almost twice as high in NAFLD/NASH patients carrying HCC than in those with NAFLD/NASH‐related cirrhosis. Further analysis of 30 NAFLD/NASH‐related HCC specimens by immunohistochemistry (IHC) revealed positive staining for OSM in liver cancer cells. The intensity of OSM immunostaining progressively increased in relation to the Edmondson–Steiner grading of HCC (Figure 1B). When NAFLD/NASH patients carrying HCC were divided according to the Barcelona Clinic Liver Cancer (BCLC) staging system [37], we observed that those in the intermediate/advanced HCC stages (classes B/C/D, respectively) had levels of circulating OSM significantly higher than those at early stages of HCC with preserved liver function (classes 0/A) (Figure 1C). In addition, the patients with a poorer prognosis and higher mortality exhibited the highest serum OSM levels at the time of diagnosis (supplementary material, Figure S1), with a trend for lower survival quite close to the significance level (Figure 1D). Interestingly, comparing circulating OSM levels in NAFLD/NASH‐related HCC patients with those in 51 patients with HCC developed on a different background (supplementary material, Table S3), it was clear that serum OSM was significantly higher in NAFLD/NASH‐HCC patients than in those with other etiologies (supplementary material, Figure S2A), particularly those with HCC developed on the background of HCV or HBV chronic infection (supplementary material, Figure S2B).

### OSM is overexpressed in experimental HCC arising in a NASH background

In order to further explore the relevance of OSM in the development of NAFLD‐related HCCs, we next studied mice with experimental NASH induced by feeding a choline‐deficient l‐amino acid‐defined (CDAA) diet [29] as well as mice with HCC originating in a NASH background using the DEN/CDAA carcinogenic protocol (supplementary material, Figure S3A) [28]. Hepatic levels of transcripts for *OSM* and its receptor (*OSMβR*, *OSMR*) were increased in rodents with NASH and were further upregulated in tumors induced by DEN/CDAA (supplementary material, Figure S3B,C). Although no significant changes in *OSM* and *OSMβR* transcript levels were observed between tumor and peri‐tumoral tissue, these transcript levels were once again significantly increased, compared with the livers of mice fed the CDAA diet (supplementary material, Figure S3D,E). Moreover, *Cdh5* (VE‐cadherin) transcripts were increased in the livers of mice with NASH and fibrosis and were even more relevant in HCC nodules, in agreement with an angiogenic switch in proliferating endothelial cells [38] (supplementary material, Figure S4A). Of note, a significant positive correlation between *OSM* and *Cdh5* transcripts was also found (supplementary material, Figure S4B), supporting the notion that OSM can exert a pro‐angiogenic action *in vivo*. In addition to VE‐cadherin, we also found a significant increase in the transcript levels of angiopoietin‐2 (*Ang2*) and *Tie2* in the livers of DEN/CDAA mice, compared with CDAA or CSAA (supplementary material, Figure S4C,D). As expected, no significant changes were detected for angiopoietin‐1 (*Ang1*) transcripts (supplementary material, Figure S4E).

### OSM acts as a pro‐angiogenic mediator

**Figure 2:**
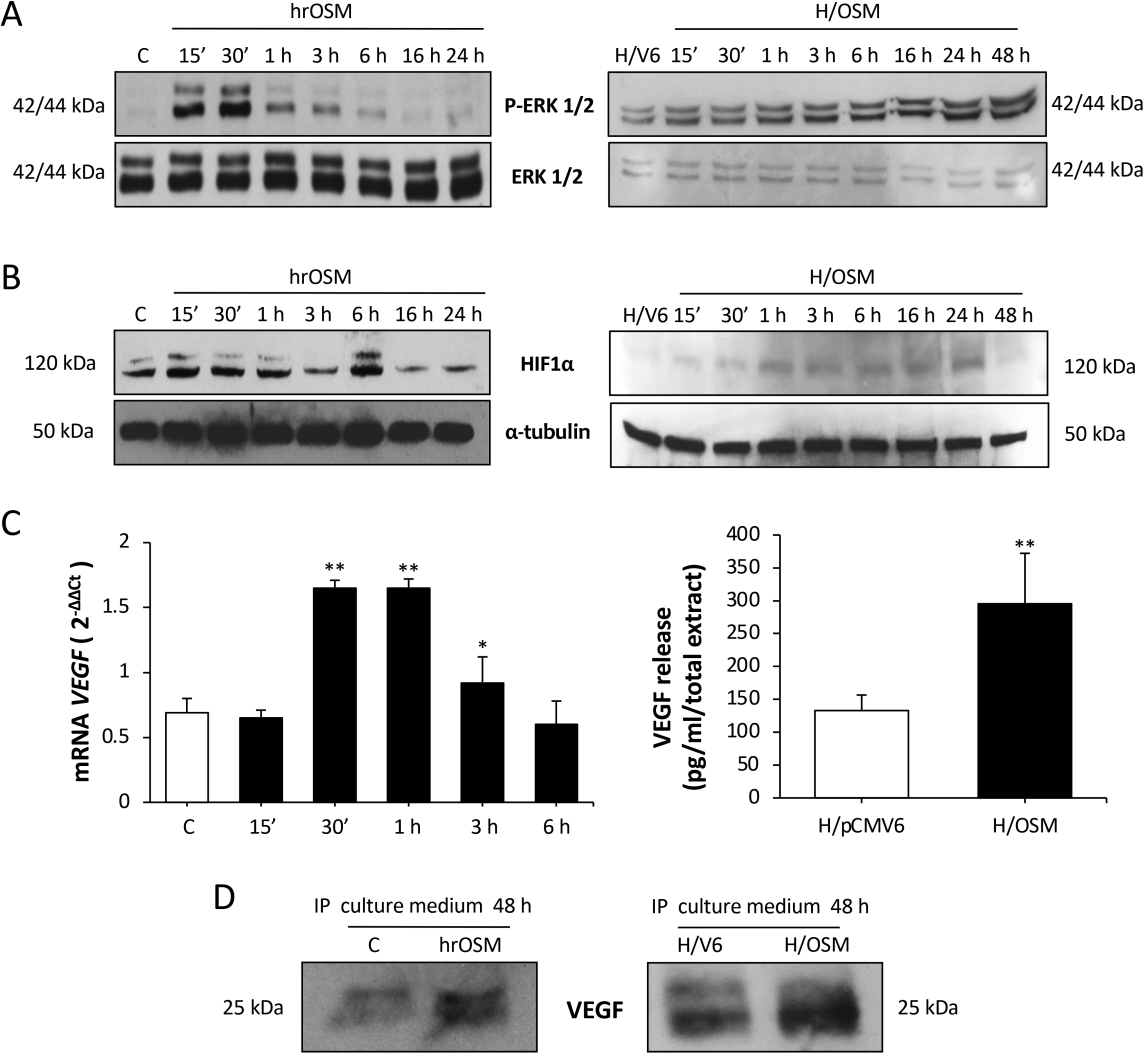
OSM upregulates VEGF expression and release. (A, B) Western blotting time course analysis of (A) P‐ERK1/2 and (B) HIF‐1α on total extracts obtained from HepG2 naïve cells exposed to hrOSM (left panel) or H/OSM (right panel). Equal loading was evaluated by re‐probed membranes for total ERK or α‐tubulin. (C) RT‐qPCR analysis of *VEGF* mRNA levels in HepG2 naïve cells exposed to hrOSM (left panel) or ELISA assay to quantify human VEGF released by H/OSM (right panel). (D) Western blotting analysis of VEGF released in culture medium by HepG2 naïve cells exposed to hrOSM (left panel) or by H/OSM (right panel).

**Figure 3:**
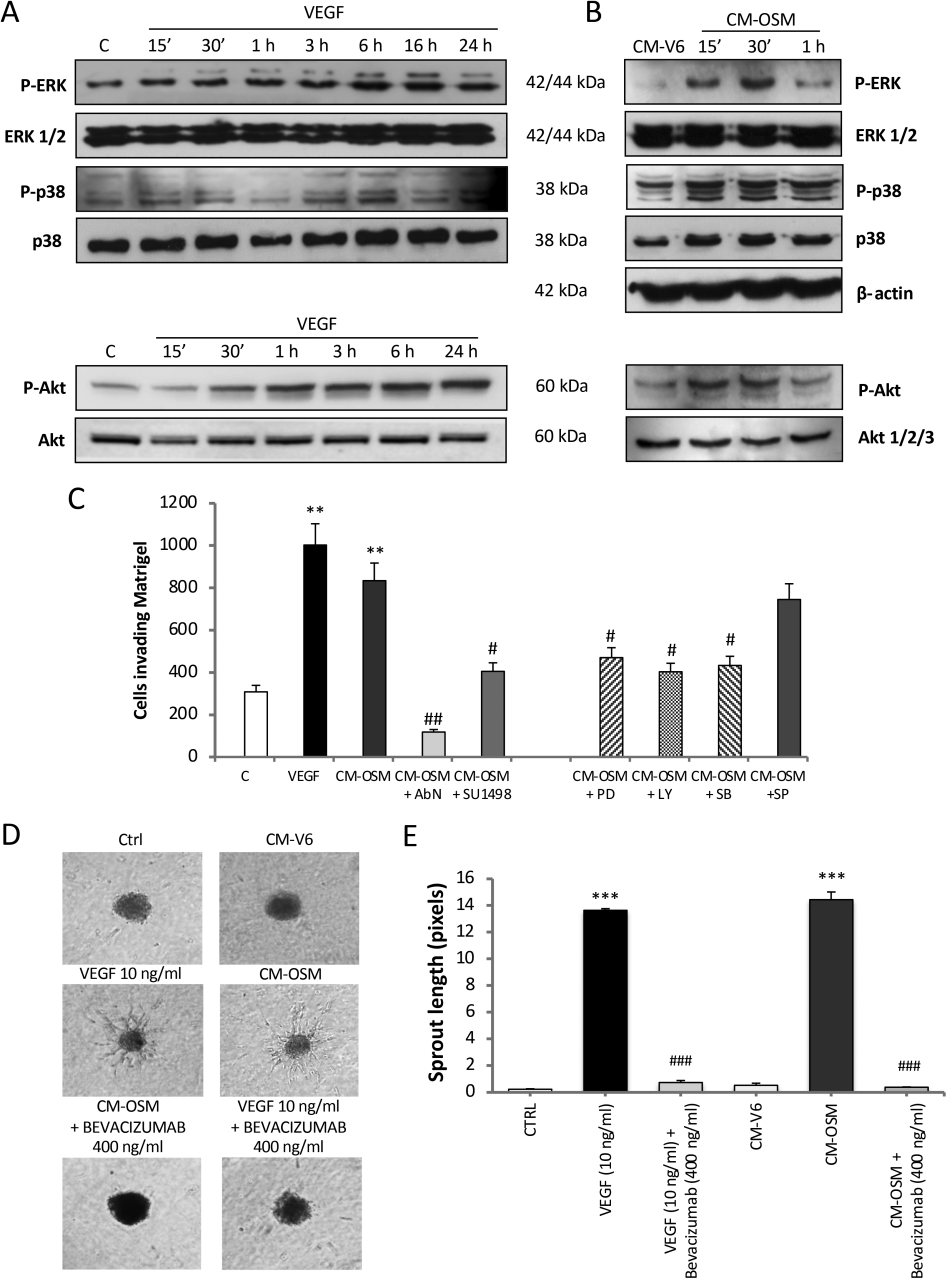
OSM‐dependent release of VEGF sustains increased invasiveness and neo‐angiogenesis. (A, B) Western blotting time course analysis of P‐ERK1/2, P‐p38, and P‐Akt (A) in HepG2 naïve cells exposed to VEGF (10 ng/ml) and (B) in HepG2 naïve cells exposed to conditioned medium collected by H/pCMV6 (CM‐V6) or H/OSM cells (CM‐OSM) after 48 h. Equal loading was evaluated by re‐probing membranes for total ERK, p38, and Akt 1/2/3. (C) Matrigel invasion assay using Boyden's chambers performed on HepG2 naïve cells exposed to conditioned medium collected by H/OSM cells after 48 h. In some conditions, HepG2 cells were pretreated with specific pharmacological inhibitors against ERK1/2 (PD, PD98059, 30 μm), PI‐3K (LY, LY294002, 10 μm), p38MAPK (SB, SB203580, 10 μm), and JNK (SP, SP600125, 20 μm) signaling pathways as well as with neutralizing antibody raised against VEGFR2 (AbN, 0.045 mg/ml) or of a specific inhibitor of VEGFR2 (SU1498, 2 μm). Data in bar charts are expressed as means ± SD of three independent experiments (**p < 0.01 versus control; ^#^ *p* < 0.05 and ^##^ *p* < 0.01 versus CM‐OSM). (D) Phase contrast analysis of sprouting spheroid assay performed with 3D collagen‐embedded HUVEC spheroids exposed to conditioned medium collected by H/pCMV6 (CM‐V6) or by H/OSM (CM‐OSM) cells after 48 h. VEGF, 10 ng/ml was used as a positive control. In some conditions, HUVEC spheroids were incubated also with bevacizumab. (E) Quantification of HUVECs sprouting. Data in bar charts are expressed as means ± SD of three independent experiments (***p < 0.001 versus CTRL; ^###^ *p* < 0.001 versus relative control condition).

Based on these data, we next established whether OSM may act as a pro‐angiogenic mediator, using HepG2 cells exposed to human recombinant OSM (hrOSM) as well as HepG2 cells stably transfected to overexpress OSM (H/OSM). OSM was found to activate ERK1/2 (Figure 2A), and to induce upregulation of HIF‐1α (Figure 2B), resulting in increased expression of the vascular endothelial growth factor‐A (*VEGF*) gene (Figure 2C, left panel) and higher release of VEGF in the culture medium (Figure 2C, right panel and Figure 2D). Treatment of HepG2 cells with conditioned medium collected from H/OSM resulted in activation of the MAPK/p38, PI‐3K/Akt, and Ras/ERK pathways (Figure 3A,B), similar to the effects of recombinant VEGF [39, 40, 41]. Therefore, we assessed the possible contribution of VEGF in mediating the ability of OSM‐stimulated HepG2 to invade across a basement membrane‐like matrix. Cell invasion was abolished by pretreatment with a blocking antibody targeting VEGF receptor 2 (VEGFR2, AbN), using SU1498, a specific pharmacological inhibitor of VEGFR2 tyrosine‐kinase activity (Figure 3C, left panel), or pharmacologically inhibiting the MAPK/p38, PI‐3K/Akt, and Ras/ERK cascades (Figure 3C, right panel).

To investigate a direct effect of OSM on angiogenesis, we used a spheroid‐based sprouting assay with human umbilical vein endothelial cells (HUVECs) [32, 33, 42]. Phase contrast analysis (Figure 3D) and related quantification of ‘sprouting’ (Figure 3E) by HUVECs exposed to conditioned medium collected from H/OSM cells (CM‐OSM) showed a significant increase in sprouting, similar to hrVEGF‐induced sprouting (Figure 3D,E). These effects were abolished by bevacizumab, a monoclonal antibody which blocks VEGF‐mediated effects (Figure 3D,E).

### OSM promotes EMT in HCC

**Figure 4:**
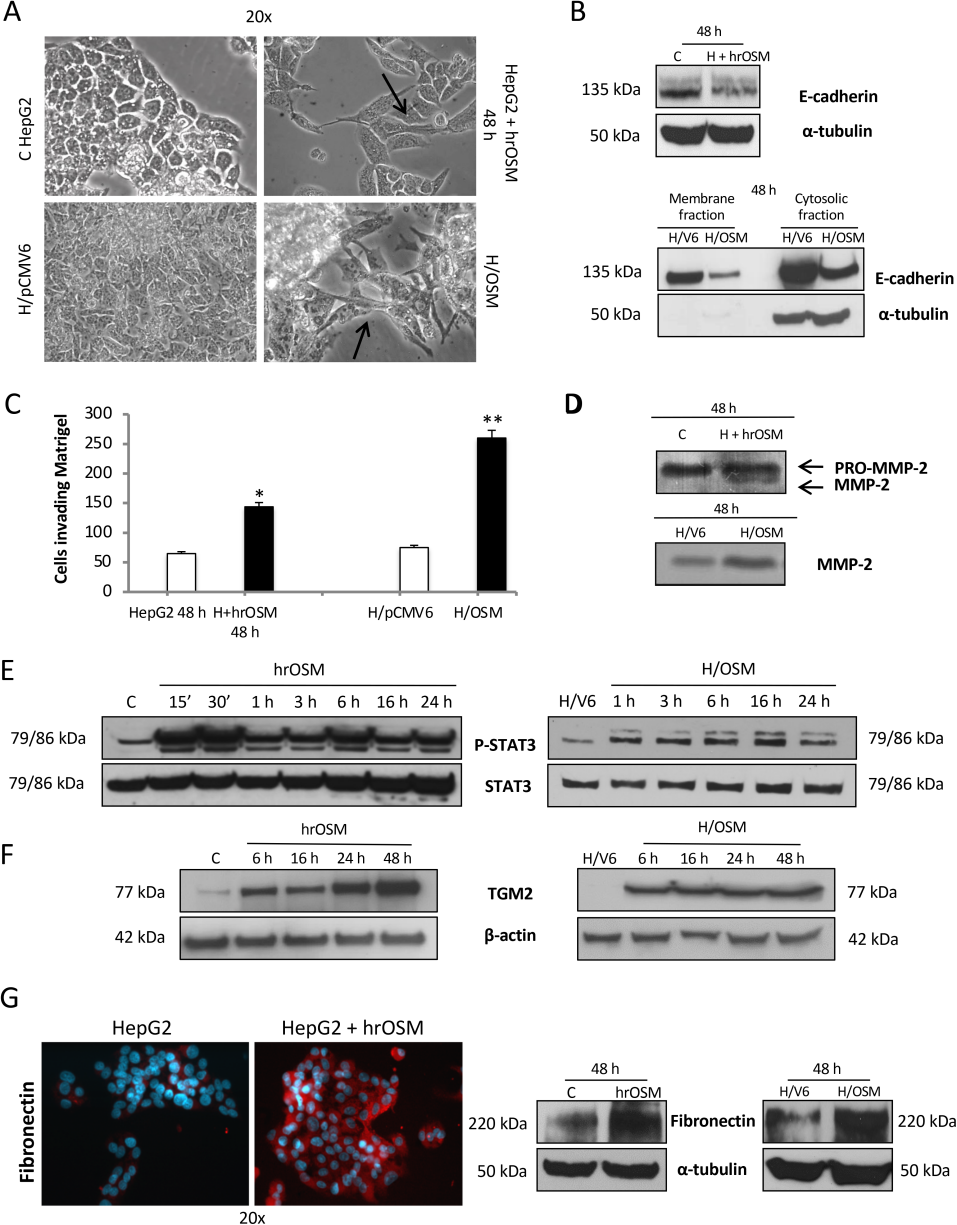
OSM induces typical EMT changes and increased invasiveness. (A) Phase contrast analysis of morphological changes into fibroblastoid‐like morphology and cellular scatter of HepG2 naïve cells exposed to hrOSM for 48 h as well as in H/OSM cells. (B) Western blotting analysis of E‐cadherin expression in total extract obtained from HepG2 naïve cells exposed to hrOSM for 48 h or in membrane and cytosolic fraction extracts from H/OSM cells. Equal loading was evaluated by re‐probing membranes for α‐tubulin (for total extract and cytosolic fraction, respectively); samples from membrane extracts were evaluated for equal loading by staining with Ponceau S solution (data not shown). (C, D) Matrigel invasion assay using (C) Boyden chambers and (D) gelatin zymography assay for detection of matrix metalloproteinase‐2 (MMP‐2) performed on HepG2 naïve cells exposed to hrOSM for 48 h or on H/OSM. (E, F) Western blotting time course analysis of P‐STAT3 and TGM2 on total extracts obtained from HepG2 naïve cells exposed to hrOSM (left panel) or in H/OSM (right panel). Equal loading was evaluated by re‐probing membranes for total STAT3 and β‐actin. (G) Expression of fibronectin in HepG2 naïve cells exposed to hrOSM for 48 h or in H/OSM evaluated by indirect immunofluorescence staining (left panel) and quantified by western blotting analysis (right panel). Equal loading was evaluated by re‐probing membranes for α‐tubulin.

Because EMT is a critical event in tumor development and progression [43, 44], we also analyzed EMT markers in HepG2 cells treated with hrOSM or in H/OSM cells. In both models, OSM induced morphologic changes typical of the EMT process, including loss of cell‐to‐cell contacts, scattering from cell clusters, and acquisition of a spindle‐shape or fibroblast‐like phenotype (Figure 4A). Consistent with this, OSM downregulated the expression of E‐cadherin on membrane fractions (Figure 4B); significantly increased the ability of cells to invade Matrigel (Figure 4C); and upregulated MMP2 levels (Figure 4D). Treatment of HepG2 cells with hrOSM or OSM overexpression also led to rapid phosphorylation and activation of STAT3 (Figure 4E). Moreover, OSM was associated with the upregulation of transglutaminase 2 (TGM2, Figure 4F), which moves to the cell membrane, where it physically interacts with integrin α5β1, and acts as a co‐receptor for fibronectin favoring cell migration and invasion [45]. Accordingly, OSM‐dependent upregulation of TGM2 was associated with increased levels of fibronectin (Figure 4G).

**Figure 5:**
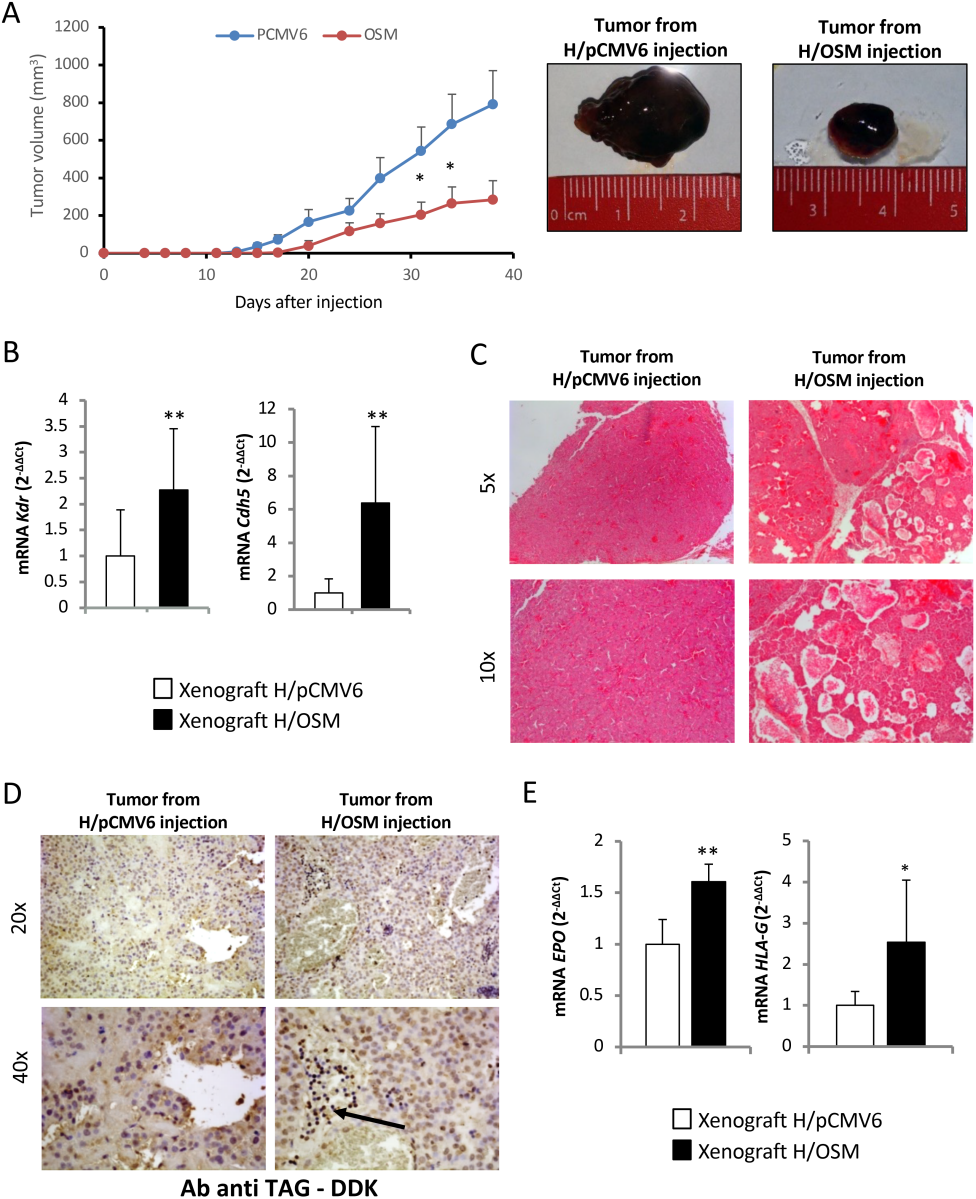
OSM increases lung metastasis in xenograft experiments. (A) Tumor volumes were measured in mice injected with H/pCMV6 and H/OSM cells and expressed as means ± SEM (*n* = 10 in the H/pCMV6 control group and *n* = 10 in the H/OSM group; *p < 0.05 versus control group). (B) RT‐qPCR analysis of mouse Flk‐1 (*Kdr*) and murine VE‐cadherin (*Cdh5*) mRNA levels in tumor masses obtained in xenograft experiments after the injection of H/pCMV6 and H/OSM cells. Data in bar charts are presented as means ± SD (**p < 0.01 versus H/pCMV6, control condition). (C, D) Hematoxylin and eosin staining and IHC analysis for DDK‐Tag performed on sections obtained from tumor xenograft masses. Original magnifications are indicated. (E) RT‐qPCR analysis of human *EPO* and *HLA‐G* mRNA levels in lung tissue from xenograft experiments. Data in bar charts are presented as means ± SD (*p < 0.05 and **p < 0.01 versus H/pCMV6, control condition).

**Figure 6:**
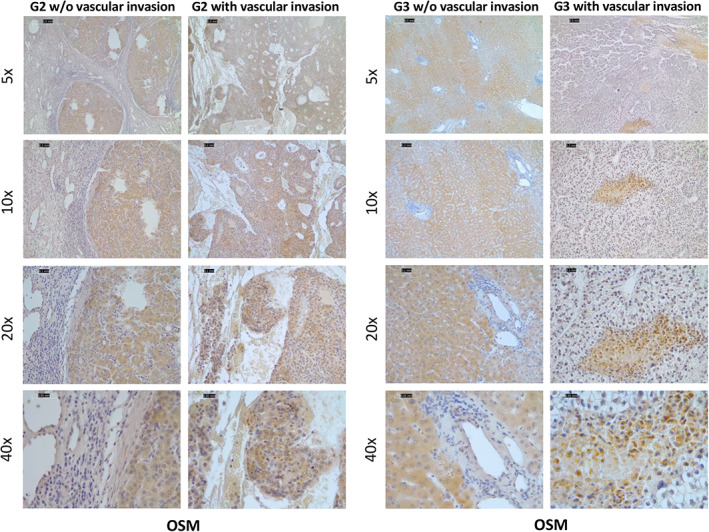
OSM expression is increased in liver cancer cells invading intra‐tumor vessels. IHC analysis for OSM was performed on paraffin‐embedded sections of G2 and G3 NAFLD/NASH‐related HCC specimens with or without vascular invasion. Original objective magnifications are indicated.

Based on the above *in vitro* results, indicating that OSM induces phenotypic changes related to EMT, we next investigated *in vivo* whether OSM may favor HCC invasiveness and metastatic potential. Xenograft experiments were conducted by injecting OSM‐overexpressing (H/OSM) or control HepG2 cells (H/pCMV6) into C57BL/6 nude mice and monitoring tumor growth for up to 42 days following inoculation. Injection of H/OSM cells resulted in tumors significantly smaller than those originating from H/pCMV6 cells (Figure 5A). To explore whether this event was associated with an increased metastatic potential, we analyzed the angiogenic features of the tumors and the possible presence of lung metastases. Tumors derived from H/OSM cells showed increased expression of the murine VEGF receptor (*Kdr*) and *Cdh5* (murine *VE‐cadherin*, Figure 5B) and the presence of more abundant and diffuse vascularization (Figure 5C). Moreover, IHC using antibodies directed against DDK‐Tag, which recognizes the transfection plasmids used in HepG2 cells, showed that immune‐positive cells were detectable in the blood vessels of tumors derived from H/OSM cells (Figure 5D). Metastatic spreading of cancer cells was evaluated by assessing the expression of human erythropoietin (*EPO*) and HLA class I histocompatibility antigen, alpha chain G (*HLA‐G*) in the lungs of mice injected with H/OSM or H/pCMV6 cells (Figure 5E). Both transcripts were significantly more abundant in mice injected with H/OSM cells, indicating that OSM overexpression confers a greater ability to generate distant metastases. In order to confirm these functional *in vivo* data in our experimental DEN/CDAA hepatocarcinogenic model, we analyzed the transcripts for selected EMT markers. As can be appreciated in supplementary material, Figure S5A–H, the mRNA levels of *SNAI1*, *MMP2*, *MMP9*, and *SNAI2* were significantly upregulated only in nodules obtained from mice undergoing the DEN/CDAA carcinogenic protocol. Even more relevant, these transcripts positively correlated with OSM expression. Moreover, by analyzing a limited number of lung samples from DEN/CDAA mice and mice fed the CSAA control diet, we detected higher levels of *EPO* transcripts in DEN/CDAA lung tissue, a finding that indicates the presence of HCC metastases (supplementary material, Figure S6). Consistent with these findings, IHC analysis of NAFLD/NASH‐related HCC specimens at stages G2 and G3 revealed the presence of OSM‐positive cancer cells invading the vessels, in contrast to what was observed in NAFLD/NASH‐related HCC specimens with the same tumor grade but without vascular invasion (Figure 6).

## Discussion

Previous studies have characterized the role of OSM in the progression of CLDs, including NAFLD, by orchestrating hypoxia‐modulated processes, parenchymal regeneration, fibrogenesis, and angiogenesis [13, 22, 23, 24]. Although OSM serum levels are elevated in cirrhotic patients [13], the actual involvement of OSM in the mechanisms of HCC progression, particularly in NAFLD/NASH patients, has not been previously investigated in detail.

In the present study, we provide evidence that OSM is overexpressed in human NAFLD/NASH‐related HCC, correlating with the disease outcome. Moreover, we show that OSM can contribute to HCC progression by switching on angiogenesis and by promoting EMT, thus favoring metastatic dissemination. In a cohort of NAFLD/NASH patients at various stages of disease evolution, including a group of subjects with NAFLD/NASH‐related HCC, circulating OSM was higher in patients carrying HCC. Immunohistochemistry studies indicated that an extensive production of OSM by HCC cells is the most likely explanation for this finding. To our knowledge, this is the first study that has specifically addressed the significance of OSM in NAFLD/NASH patients. In fact, a single study has previously reported an increase in OSM serum levels in a small cohort of HBV/HCV patients with or without HCC, without any information on the possible correlation with disease outcomes [13]. OSM upregulation was a specific feature of NAFLD/NASH‐derived HCC, since OSM serum levels were higher than those detected in patients with HCCs of non‐NAFLD etiologies, e.g. associated with viral infection or alcohol use disorder. At the time of diagnosis, circulating OSM in NAFLD/NASH‐related HCCs was particularly elevated in subjects with advanced or terminal stage tumors and was associated with a poor prognosis and higher mortality, suggesting that OSM production might be driving HCC progression and that the evaluation of OSM serum levels may be useful for estimating HCC prognosis.

At present, the precise mechanisms underlying OSM upregulation in NASH are uncertain and deserve further investigation, although it is possible that altered lipid metabolism or lipotoxicity may be involved [46]. It should be noted that when NASH‐HCCs were compared with HCCs of other etiologies, they displayed a significant enrichment of signatures related to a number of processes, including (1) bile acid and fatty acid metabolism (including cholesterol and sterol biosynthesis), (2) oxidative stress and reactive oxygen species (ROS) production, and (3) inflammation [47]. Furthermore, it has been reported that NASH‐HCCs were enriched in the Wnt/TGF‐beta class and displayed a significantly lower prevalence of the CTNNB1 molecular subclass compared with viral/alcohol‐HCCs. In other words, the S1 signature in NASH‐HCCs is related to a more aggressive and less differentiated phenotype [48], suggesting the possibility that OSM may significantly contribute to the acquisition of a more aggressive phenotype. In line with this possibility, we also provide evidence for an involvement of OSM in HCC progression. In fact, *in vitro* experiments indicated that HepG2 cells overexpressing OSM undergo EMT‐related changes such as acquisition of fibroblast‐like morphology, loss of E‐cadherin, and reduction of cell‐to‐cell adhesion [45, 49]. These changes favor migration and increased invasiveness of hepatic cancer cells, an effect in agreement with previous studies performed on breast [19], gastric [50], pancreatic [51], and cervical squamous cell cancer [20]. OSM also resulted in the stimulation of VEGF expression, at the gene and protein levels, which in turn sustains OSM‐dependent invasiveness of hepatic cancer cells, through the activation of MAPK/p38, PI‐3K/Akt and Ras/ERK1/2 signaling pathways, and contributes to angiogenesis. The functional relevance of OSM‐dependent production of VEGF is supported by experiments using spheroid‐based sprouting angiogenesis. Moreover, OSM was found to correlate with *Cdh5* transcripts in HCC nodules developing in a murine, NASH‐related, carcinogenic model. These data are consistent with previous studies indicating that OSM can induce the expression of HIF‐1α and its target genes under normoxic conditions [52], thus stimulating endothelial activation [53] and angiogenesis in prostate and endometrial cancers [54, 55].

Increased VEGF levels are found in several solid tumors [56, 57] and are associated with vascularity, metastasis, and chemotherapy resistance. Xenografting experiments described in this article indicate that injection of HepG2 cells overexpressing OSM led to the formation of a smaller tumor but with a much wider and diffuse vascularization. These results are consistent with previous studies showing that OSM can inhibit cell growth in several tumor cell lines [58, 59], inducing in parallel morphological and phenotypical alterations consistent with a more invasive phenotype [60]. More importantly, cells overexpressing OSM were detected inside blood vessels only in tumors derived from H/OSM injection, indicating the angio‐invasive and metastatic features of these cells. In agreement with this hypothesis, a higher number of metastatic cells were found in the lungs of mice receiving OSM‐overexpressing cells. These features are similar to those reported by Bolin *et al* in breast cancer cells, where the anti‐proliferative effects of OSM were associated with an increased rate of bone metastasis [18]. Taken together, these data strongly indicate that OSM determines a phenotypic switch in hepatic cancer cells, making them more invasive and capable of orchestrating the formation of new blood vessels.

A very recent study by Yang *et al* [61] showed that OSM overexpression in a DEN‐induced rat model of liver carcinogenesis resulted in a significant increase in the number of tumor nodules and shortened the overall survival of the animals. In this experimental model, OSM promoted hepatocarcinogenesis through the activation of hepatic progenitor cells (HPCs) resulting from TNF‐α and macrophage accumulation. Those authors also provided data indicating that OSM expression in the peritumoral tissues of human HCC positively correlated with poor overall survival of patients [61]. These very recent data are consistent with the hypothesis of a pro‐carcinogenic role of OSM provided by our study. However, it should be noted that the DEN model of hepatocarcinogenesis used in Yang *et al*’s study is unrelated to NASH‐induced HCC and no details were provided about the etiology of human HCC specimens analyzed [61]. Along these lines, previous studies have already outlined that OSM may have a pro‐fibrogenic role either by directly affecting activated, MF‐like, HSC [24] or by coordinating the phenotypic changes of human macrophages and HSCs [62]. Further experiments are needed to investigate whether OSM may affect in a similar way either tumor‐associated macrophages (TAMs) or cancer‐associated fibroblasts (CAFs).

In conclusion, our findings have the potential to considerably advance our understanding of the biology of hepatic cancer. The possible role of OSM as a prognostic biomarker and a target to halt HCC progression deserves further evaluation in prospective studies.

## Author contributions statement

GDM, BF, SC, EA, FM, EB, PP and PM designed the experiments, interpreted data and drafted the manuscript. SS performed the animal experiments. GDM, BF, LN, CT, SS and EN acquired and analyzed the data. MM, EN, MA, RA, SG, CR and AB contributed to the acquisition of the data. FB and SB provided substantial contributions to the conception of the study, experimental design, and interpretation of the data. MP, PP, EB and FB acquired financial support for the study. EB, CR, PP, SG, PC and CT collected samples and clinico‐pathologic data. MA performed the pathological assessments. All the authors revised and approved the final version of the manuscript.

## Supporting information


Supplementary materials and methods

**Figure S1.** OSM serum levels are potentially predictors of poor prognosis in NASH‐related HCC patients
**Figure S2.** OSM serum levels in different cohorts of HCC patients
**Figure S3.** Experimental model of hepatocarcinogenesis
**Figure S4.** *In vivo* correlation between OSM expression and angiogenesis
**Figure S5.** *In vivo* correlation between OSM expression and EMT
**Figure S6.** *In vivo* metastasis in mice undergoing liver carcinogenesis using the DEN + CDAA protocol
**Table S1.** Biochemical characteristics of NAFLD patients according to the severity of liver disease
**Table S2.** Clinical and biochemical characterization of NAFLD/NASH patients carrying HCC
**Table S3.** Clinical and biochemical characterization of mixed etiology‐related HCC patients (alcohol, HCV, HBV, autoimmune, and metabolic)
**Table S4.** Oligonucleotide primers used for qPCR
