# Supplementary material for: Oncostatin M is overexpressed in NASH‐related hepatocellular carcinoma and promotes cancer cell invasiveness and angiogenesis
Source: J Pathol. 2022 Mar 7;257(1):82–95. doi: 10.1002/path.5871 (PMC9315146; doi:10.1002/path.5871)
Supplement: Supplementary file 1 — Supplementary materials and methods [file PATH-257-82-s002.docx]

**Oncostatin M is overexpressed in NASH-related hepatocellular carcinoma and promotes cancer cell invasiveness and angiogenesis**

G Di Maira, B Foglia, *et al.* DOI: 10.1002/path.5871

**Supplementary materials and methods**

Reference numbers refer to the main text reference list

*Materials and laboratory reagents*

The choline-deficient amino acid-refined (CDAA) diet as well as the related control diets and methionine choline-supplemented (MCS) diet were provided by Laboratorio Dottori Piccioni srl (Gessate, Milano, Italy). The kits for RNA reverse transcription and for quantitative real-time PCR were purchased from Bio-Rad Laboratories (Berkeley, CA, USA). Enhanced chemiluminescence (ECL) reagents and nitrocellulose membranes (Hybond-C extra) were from Amersham Pharmacia Biotech Inc (Piscataway, NJ, USA). Human recombinant oncostatin M (OSM) was from Petrotech (Rocky Hill, CT, USA). The monoclonal antibodies against p-ERK1/2 (sc-7383), fibronectin (sc-8422), and transglutaminase-2 (sc-48370), and the polyclonal antibodies for p-STAT3 (sc-8059), STAT3 (sc-8019), ERK1/2 (sc-292838), p-Akt1/2/3 (sc-7985-R), Akt1/2/3 (sc-8312), VEGF (sc-152), and E-cadherin (sc-7870) were from Santa Cruz Biotechnology (Dallas, TX, USA). Polyclonal antibodies for p-p38 (#9211) and p38 (#9212) were from Cell Signaling Technology (Danvers, MA, USA). The monoclonal antibodies for β-actin (A5441) and α-tubulin (T9026) were from Merck-Sigma Aldrich (St Louis, MO, USA). The polyclonal antibody for HIF-1α (NB100-479) was from Novus Biologicals (Cambridge, UK); PD98059, SB202190, SP600125, LY294002, and SU1498 were from Calbiochem (La Jolla, CA, USA). Mayer’s Hematoxylin Solution and all other reagents of analytical grade as well as primers for RT-qPCR were from Merck-Sigma Aldrich; Boyden chambers were from Neuro Probe, Inc (Gaithersburg, MD, USA). The monoclonal neutralizing antibody against Flk-1 was obtained from ImClone (New York, NY, USA). The HiPerfect Transfection Reagent was from Qiagen (Hilden, Germany); Lipofectamine 2000 was from Invitrogen-Life Technologies (Carlsbad, CA, USA); and plasmid DNA purification NucleoBond XtraMIDI was from Macherey-Nagel (Düren, Germany). TrueORF Gold Clones – Expression validated cDNA Clones (pCMV6-Entry, mammalian vector with C-terminal Myc-DDK Tag and Oncostatin M (OSM) Human Myc-DDK-tagged ORF Clone) as well as anti-DDK (FLAG) monoclonal antibody were from OriGene Technologies, Inc (Rockville, MD, USA). The cell proliferation ELISA BrdU Colorimetric assay kit was from Roche (from Merck-Sigma Aldrich).

*Protein extraction*

For total extracts, HepG2 naïve cells or H/V6 and H/OSM cells were seeded in normoxic conditions to obtain the desired sub-confluence level (65–70%). HepG2 cells were treated with human recombinant OSM for the indicated times. Concerning HepG2-transfected cells, after 20 h from plating, a time course analysis was performed from 15 min up to 48 h. At the indicated time points, collected cells were suspended in lysis buffer containing 20 mm Tris/HCl (pH 7.4), 150 mm NaCl, 5 mm ethylenediaminetetraaceticacid (EDTA), 0.1 mm phenylmethyl-sulfonyl fluoride (PMSF), 0.05% aprotinin, and 0.1% IGEPAL, and then incubated for 30 min at 4 °C. The suspension was centrifuged for 25 min at 12 000 rpm and the resulting supernatant was saved as the total protein extract.

For membrane-associated fraction extracts, cells were seeded in 75 cm^2^ dishes. At the indicated time points, collected cells were suspended in hypotonic buffer containing 10 mm Tris (pH 7.4), 0.2 mm MgCl_2_, 2 µg/ml pepstatin A, 2 µg/ml leupeptin, and 100 µg/ml PMSF for 30 min on ice, and then homogenized with a cell scraper. The homogenates were centrifuged for 35 min at 20 750 rpm; the resulting precipitates were suspended in sample buffer 2X and analyzed as the membrane-associated fraction, while supernatants were analyzed as the cytosolic fraction.

In some experiments, protein levels in culture medium obtained from HepG2 naïve cells treated with human recombinant OSM (hrOSM) or H/OSM for the desired time were evaluated by an immunoprecipitation procedure. For immunoprecipitation experiments, cells were seeded in 75 cm^2^ dishes and treated with human recombinant OSM (HepG2 cells) or cultured (H/pCMV6 and H/OSM cells) for the indicated times. At the indicated time point, collected cells were centrifuged (5 min, 1500 rpm) and supernatants were collected and supplemented with protease and phosphatase inhibitors (50 µg/ml pepstatin, 50 µg/ml leupeptin, 10 µg/ml aprotinin, 1 mm phenylmethylsulfonyl fluoride, 1 mm Na_3_VO_4_). Collected medium samples (1 ml) were then incubated with protein A-Sepharose (Merck-Sigma Aldrich) and anti-VEGF antibody (1:50) for 2 h at 4 °C. Immunoprecipitates were washed four times with lysis buffer, and proteins were solubilized under reducing conditions, separated by SDS-PAGE (10%), transferred to a nitrocellulose membrane, and incubated with the VEGF-A primary antibody.

*Spheroid-based sprouting angiogenesis* in vitro *assay*

HUVECs were suspended at a density of 4000 cells/ml in culture medium containing 20% Methocel stock solution (12 mg/ml carboxymethyl cellulose in M199, from Merck-Sigma Aldrich) and 20% FCS, as described previously [32,33]. In brief, 800 cells were seeded into non-adherent round-bottom 96-well plates (BD Biosciences, [Franklin Lakes, NJ, USA](https://www.google.com/search?rlz=1C1EJFA_enIT673IT673&q=Franklin+Lakes&stick=H4sIAAAAAAAAAOPgE-LUz9U3MCozKDZV4gAxK7ILK7S0spOt9POL0hPzMqsSSzLz81A4VhmpiSmFpYlFJalFxYtY-dyKEvOyczLzFHwSs1OLd7AyAgA3qgWwVwAAAA&sa=X&ved=2ahUKEwjMsrCe9Kn1AhW_if0HHZ2nCRcQmxMoAXoECCEQAw)) and cultured overnight at 37 °C. The spheroids were harvested by gently pipetting and centrifuged at 300 × *g* for 15 min. The spheroids were then resuspended in (i) 200 μl of M199 medium containing 40% FCS, 1.2% (v/w) methylcellulose without or with 10 ng/ml VEGF-A (R&D Systems, Minneapolis, MN, USA) used as a positive control or (ii) 100 μl of M199 medium containing 40% FCS, 1.2% (v/w) methylcellulose plus 100 μl of conditioned medium collected from H/pCMV6 or H/OSM cells. In some experimental conditions, humanized anti-VEGF-A monoclonal antibody (bevacizumab 400 ng/ml) was tested. In all wells, an equal volume of a collagen solution [7 volumes of collagen from rat tail, 1 volume of M199, 1 volume of 0.1 m NaOH, 1 volume of 0.2 m *N*-(2-hydroxyethyl)piperazine-*N*′-(2-ethanesulfonic acid) (HEPES buffer, pH 7.3)] was added. Spheroids were then seeded in 96-well plates and incubated at 37 °C. After 24 h, phase-contrast images were captured by using a CCD Hamamatsu ORCA camera (Hamamatsu Photonics Italia, Arese, Italy) linked to an inverted microscope (model DM IRB HC; Leica Microsystems, Heerbrugg, Switzerland). Sprout length was quantified using ImageJ image analysis software (National Institutes of Health, Bethesda, MD, USA).
