# Supplementary material for: Oncostatin M is overexpressed in NASH‐related hepatocellular carcinoma and promotes cancer cell invasiveness and angiogenesis
Source: J Pathol. 2022 Mar 7;257(1):82–95. doi: 10.1002/path.5871 (PMC9315146; doi:10.1002/path.5871)
Supplement: Supplementary file 2 — Figure S1. OSM serum levels are potentially predictors of poor prognosis in NASH‐related HCC patients Figure S2. OSM serum levels in different cohorts of HCC patients Figure S3. Experimental model of hepatocarcinogenesis Figure S4. In vivo correlation between OSM expression and angiogenesis Figure S5. In vivo correlation between OSM expression and EMT Figure S6. In vivo metastasis in mice undergoing liver carcinogenesis using the DEN + CDAA protocol Table S1. Biochemical characteristics of NAFLD patients according to the severity of liver disease Table S2. Clinical and biochemical characterization of NAFLD/NASH patients carrying HCC Table S3. Clinical and biochemical characterization of mixed etiology‐related HCC patients (alcohol, HCV, HBV, autoimmune, and metabolic) Table S4. Oligonucleotide primers used for qPCR [file PATH-257-82-s001.docx]

**Oncostatin M is overexpressed in NASH-related hepatocellular carcinoma and promotes cancer cell invasiveness and angiogenesis**

G Di Maira, B Foglia, *et al*. *J Pathol* DOI: 10.1002/path.5871

**Supplementary Figures S1–S6**

**Supplementary Tables S1–S4**


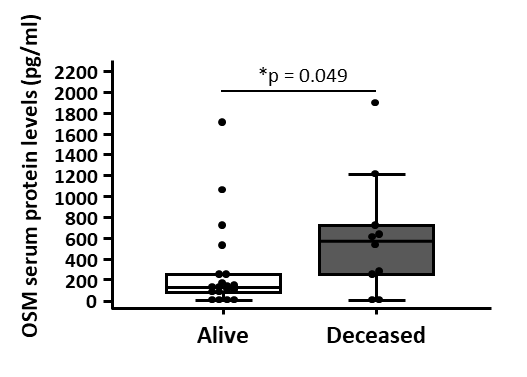


**Figure S1.** **OSM serum levels are potentially predictors of poor prognosis in NASH-related HCC patients.** OSM serum protein levels of NASH-related HCC patients according to survival. Data are reported as median and 95% CI of the median (**p* < 0.05).

**
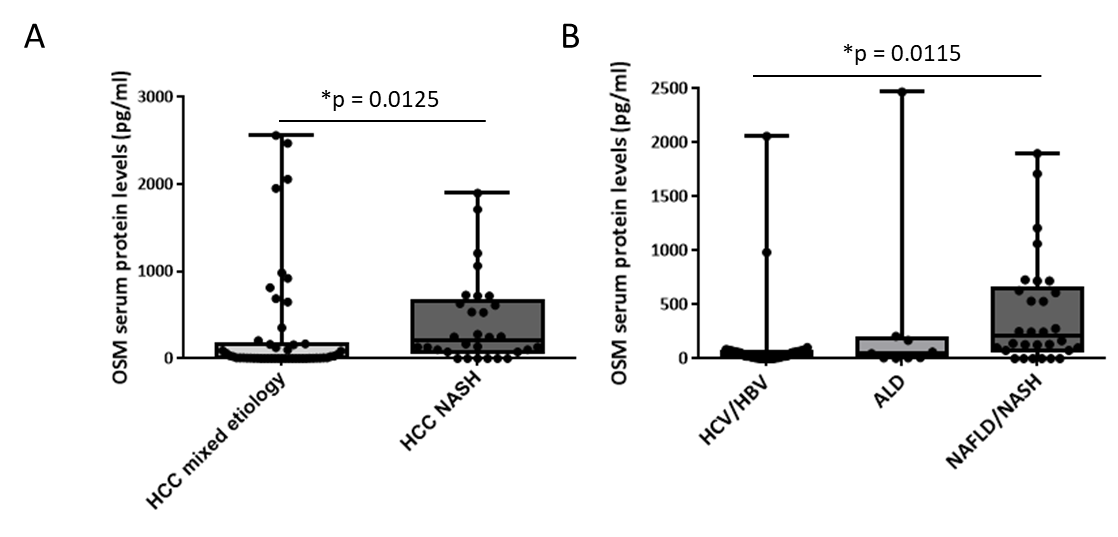
**

**Figure S2. OSM serum levels in different cohorts of HCC patients.** (A) Comparison of OSM protein levels in HCC patients of mixed etiology versus NASH-related HCC patients. Mann–Whitney test of frequency distribution data (**p* < 0.05). (B) OSM serum levels stratified in relation of etiology. One-way ANOVA test with Tukey’s correction for multiple comparisons (**p* < 0.05). Boxes include the values within the 25th and 75th percentiles, whereas the horizontal bars represent the medians. The extremities of the vertical bars comprise the minimum and the maximum value.


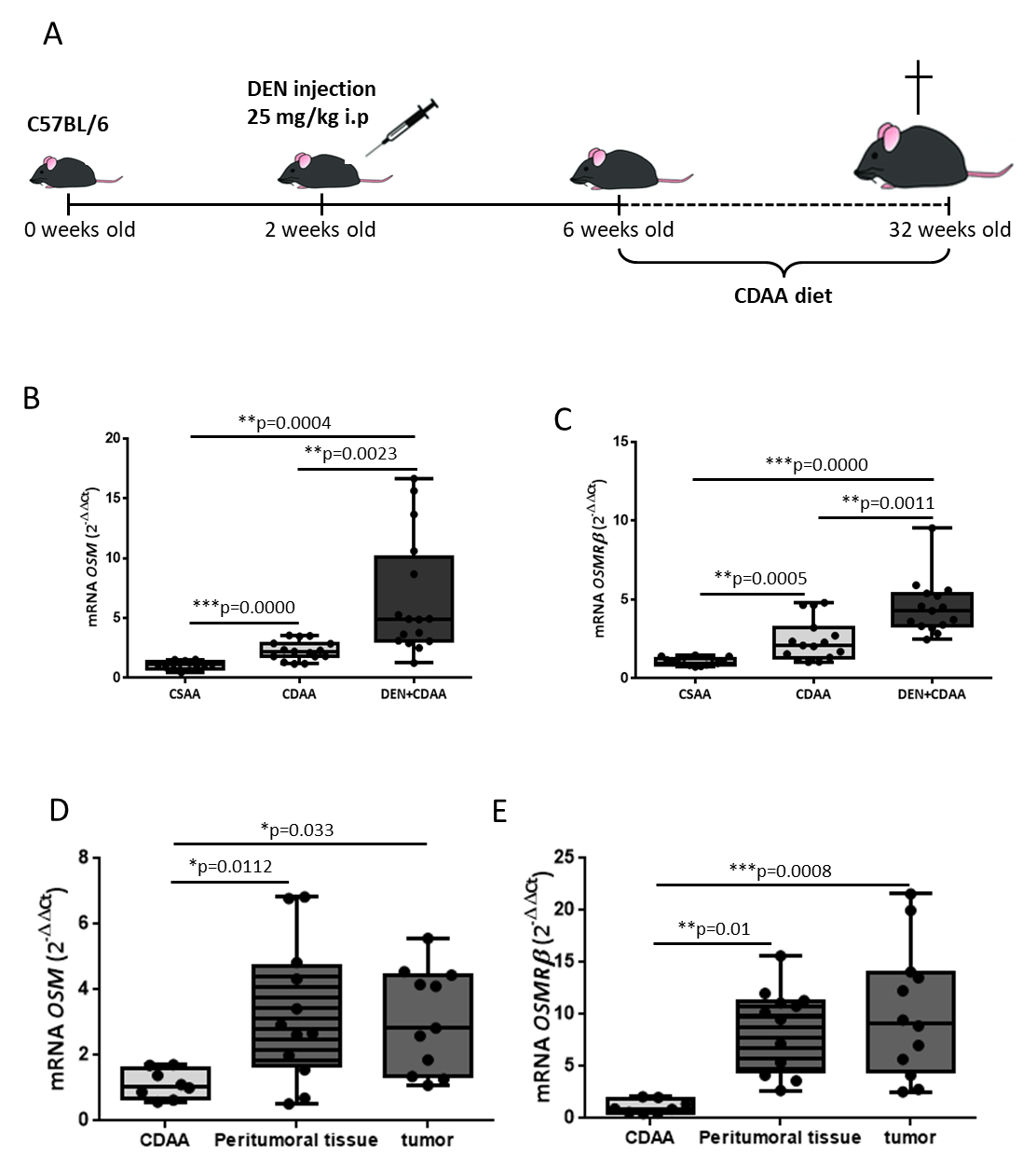


**Figure S3. Experimental model of hepatocarcinogenesis.** (A) Wild-type C57BL/6 mice were submitted to (a) DEN + CDAA carcinogenic protocol, (b) CDAA diet or (c) CSAA control diet. (B, C) qPCR analysis of (B) *OSM* and (C) *OSMβR* transcripts in wild-type C57BL/6 mice submitted to the DEN + CDAA protocol or fed the CDAA diet compared with corresponding littermates fed the CSAA control diet. (D, E) RT-qPCR analysis of (D) *OSM* and (E) *OSMβR* transcript levels in peritumoral tissue and nodules of mice submitted to the DEN + CDAA protocol compared with mice fed the CDAA diet. Statistical analysis was performed with a Kruskal–Wallis test of one-way ANOVA data with Dunn’s correction for multiple comparisons of frequency distribution data (*p<0.05; ***p* < 0.01 and ****p*< 0.001). Boxes include the values within the 25th and 75th percentiles, whereas the horizontal bars present the medians. The extremities of the vertical bars comprise the minimum and the maximum value.

**
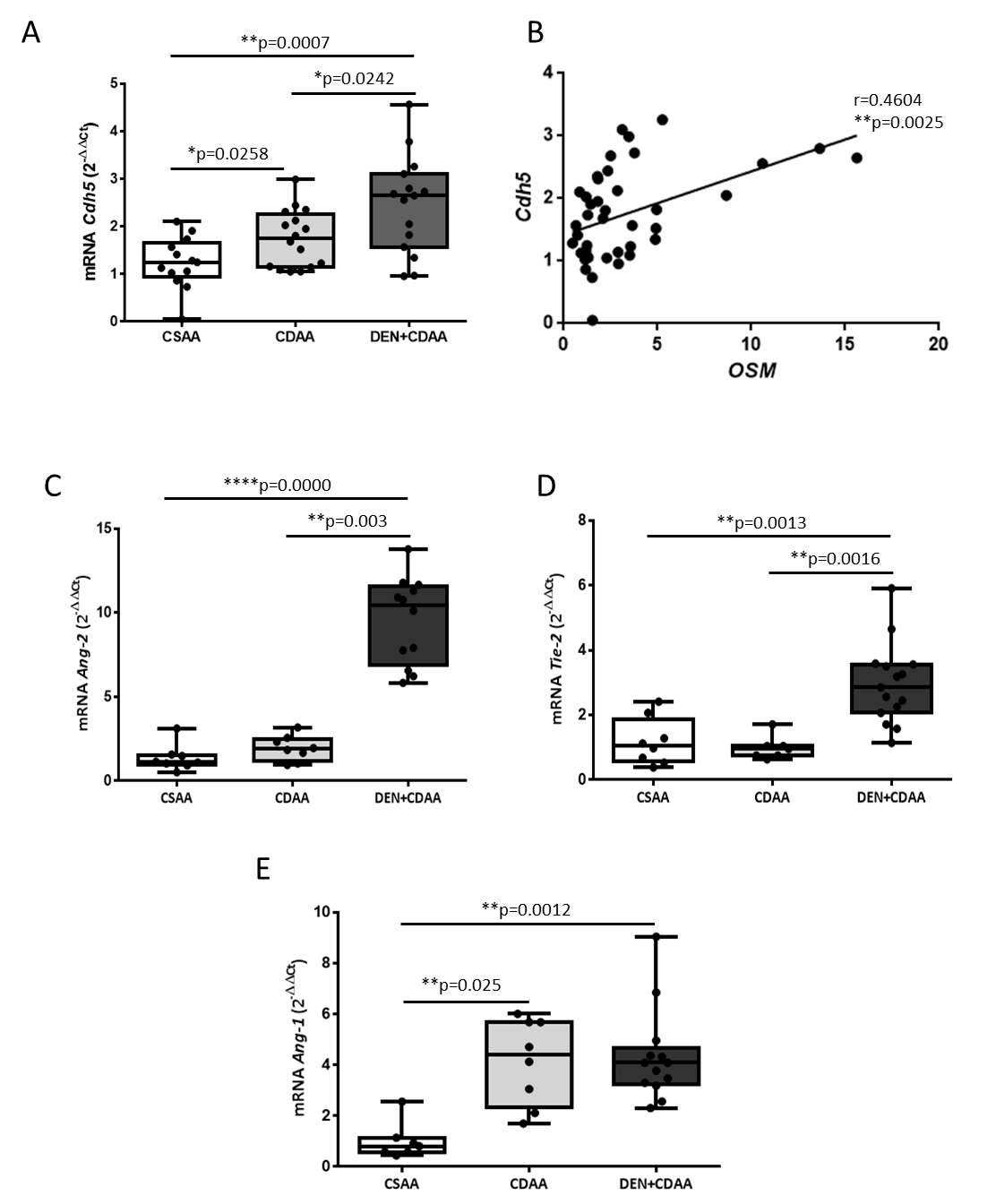
**

**Figure S4. *In vivo* correlation between OSM expression and angiogenesis.** (A, C, D, E) RT-qPCR analysis of (A) *Cdh5*, (C) *Ang2*, (D) *Tie2*, and (E) *Ang1* transcript levels in wild-type C57BL/6 mice submitted to DEN + CDAA or fed the CDAA diet compared with corresponding littermates fed the CSAA control diet. Statistical analysis was performed using a Kruskal–Wallis test of one-way ANOVA data with Dunn’s correction for multiple comparisons of frequency distribution data (*p<0.05; ***p*< 0.01 and *****p*< 0.001). Boxes include the values within the 25th and 75th percentiles, whereas the horizontal bars present the medians. The extremities of the vertical bars comprise the minimum and the maximum value. (B) Positive correlation between *Cdh5* and *OSM* transcript levels. Statistical analysis was performed using Pearson’s correlation (Pearson’s *r* 0.4604, ***p* < 0.01).


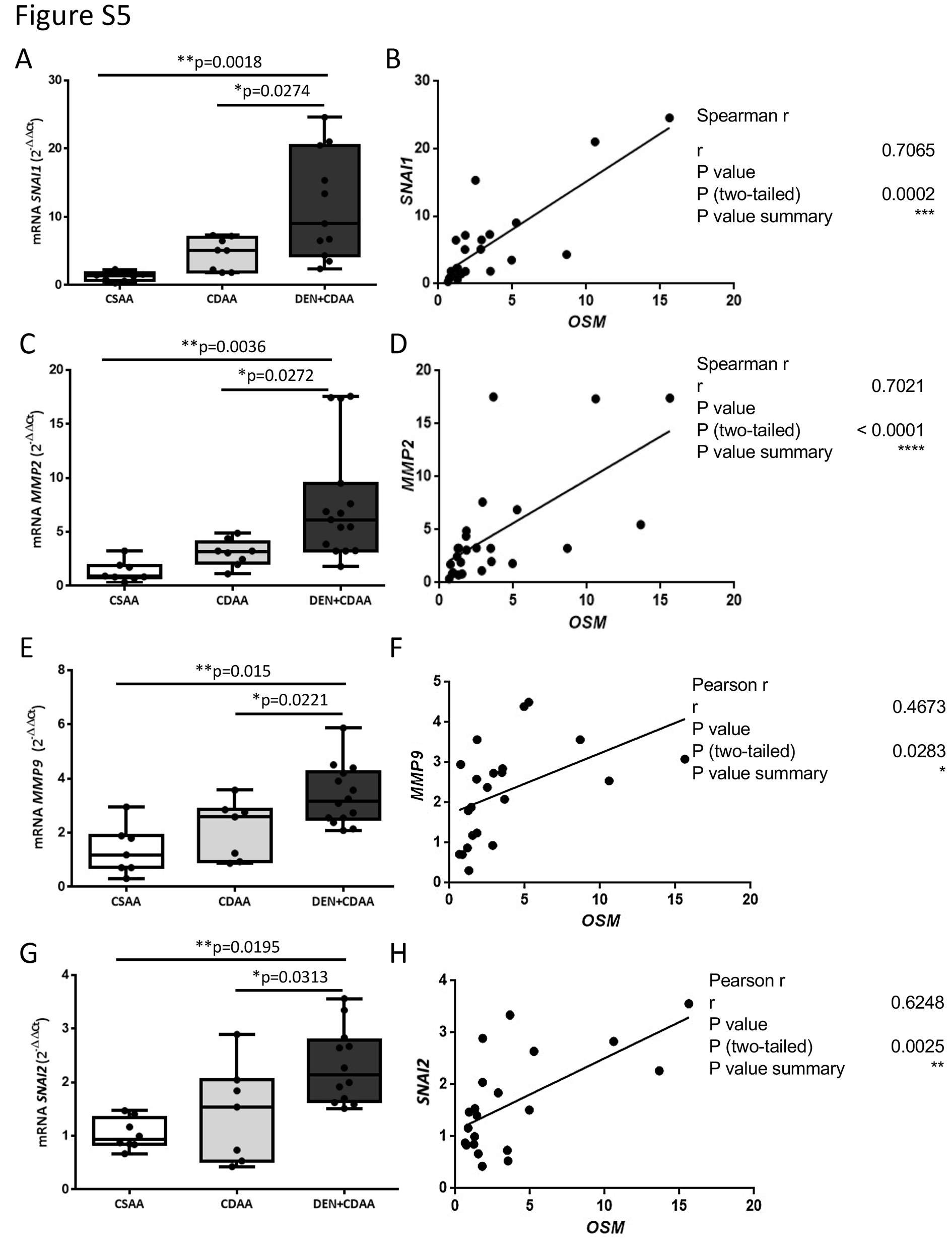


**Figure S5.** ***In vivo* correlation between OSM expression and EMT.** (A, C, E, G) RT-qPCR analyses for (A) *SNAI1*, (C) *MMP2*, (E) *MMP9*, and (G) *SNAI2* transcript levels in wild-type C57BL/6 mice submitted to DEN + CDAA or fed the CDAA diet compared with corresponding littermates fed the CSAA control diet. Statistical analysis was performed using a Kruskal–Wallis test of one-way ANOVA data with Dunn’s correction for multiple comparisons of frequency distribution data (*p<0.05; ***p* < 0.01). Boxes include the values within the 25th and 75th percentiles, and the horizontal bars present the medians. The extremities of the vertical bars comprise the minimum and the maximum value. (B, D, F, H) Positive correlation between EMT markers (*SNAI1*, *MMP2*, *MMP9*, and *SNAI2*) and *OSM* transcript levels. Statistical analysis was performed using Spearman or Pearson correlation, as indicated.

**
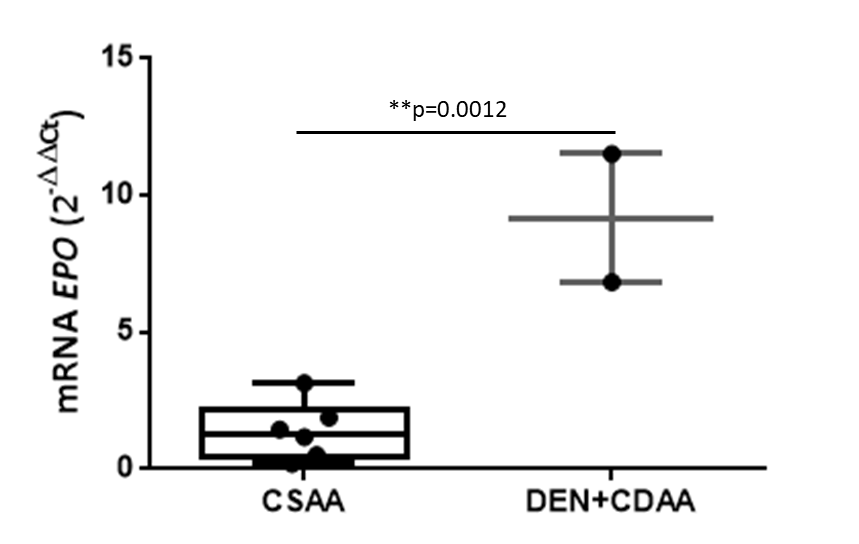
**

**Figure S6. *In vivo* metastasis in mice undergoing liver carcinogenesis using the DEN + CDAA protocol**. RT-qPCR analysis of *EPO* transcript levels in lung tissue from wild-type C57BL/6 mice submitted to the DEN + CDAA protocol compared with corresponding littermates fed the CSAA control diet. Mann–Whitney test of frequency distribution data (**p<0.01 versus control condition).

**Table S1**. Biochemical characteristics of NAFLD patients according to the severity of liver disease

| **Variable** | **All (*N* = 101)** | **Simple steatosis (*N* = 6)** | **NASH**  **(*N* = 15)** | **Cirrhosis**  **(*N* = 32)** | ***P* value** |
| --- | --- | --- | --- | --- | --- |
| Age (years) | 53 | 49 | 45 | 61 | **<0.001** |
| BMI (kg/m^2^) | 29.3 | 26.9 | 29.1 | 30.6 | 0.243 |
| AST (U/l) | 32 | 29 | 30 | 36 | 0.316 |
| ALT (U/l) | 46 | 49 | 51 | 37 | 0.074 |
| γ-GT (U/l) | 55 | 52 | 45 | 85 | **0.012** |
| Hb (g/dl) | 15 | 15 | 15.1 | 14.9 | 0.651 |
| Platelets | 214 | 218 | 224 | 191 | **0.002** |
| Albumin (g/l) | 4.3 | 4.4 | 4.4 | 4.2 | 0.143 |
| Total bilirubin (mg/dl) | 0.8 | 0.8 | 0.6 | 0.8 | 0.145 |
| Fasting glucose (mg/dl) | 94 | 96 | 92 | 102 | **0.012** |
| Fasting insulin (U/l) | 13.8 | 11.7 | 13 | 18.3 | 0.072 |
| HOMA-IR | 3.5 | 2.6 | 3 | 5.8 | **0.021** |
| Triglycerides (mg/dl) | 123 | 126 | 116 | 129 | 0.442 |
| Total cholesterol (mg/dl) | 194 | 201 | 195 | 184 | 0.273 |
| HDL-cholesterol (mg/dl) | 46 | 42 | 48 | 41 | 0.590 |

Data are reported as median and interquartile range. Differences between groups were assessed using the Kruskal–Wallis non-parametric test. *p* < 0.05 (shown in bold) was considered statistically significant.

**Table S2.** Clinical and biochemical characterization of NAFLD/NASH patients carrying HCC

| ***Demographic data*** | |
| --- | --- |
| Number of patients (male/female) | 30 |
| Age (years) | 67 |
| BMI (kg/m^2^) | 28.2 |
| ***Clinical data*** | |
| CHILD | A (83.3%); B/C (10%) |
| BLCL | 0-A (70%), B-C-D (30%) |
| MELD | 5 |
| ***Biochemical data*** | |
| AST (U/l) | 38.4 |
| ALT (U/l) | 33.8 |
| γ-GT (U/l) | 122 |
| INR | 1.2 |
| Bilirubin (U/l) | 1.3 |
| AFP (ng/ml) | 50.3 |
| Albumin (g/l) | 3.7 |
| ***Oncological data*** | |
| Number of nodules | 1.9 |
| Dimension (mm) | 31.1 |

The values are expressed as median and inter-quartile range (IQR). For histological scores the range of variability is included.

BMI, body mass index; AST, alanine aminotransferase; ALT, aspartate aminotransferase; γ-GT, gamma-glutamyl transpeptidase.

**Table S3.** Clinical and biochemical characterization of mixed etiology-related HCC patients (alcohol, HCV, HBV, autoimmune, and metabolic)

| ***Demographic data*** | |
| --- | --- |
| Number of patients (male/female) | 51 |
| Age (years) | 63 |
| BMI (kg/m^2^) | 28.1 |
| ***Clinical data*** | |
| CHILD | A (50.9%); B/C (33.3%); ND (15.6%) |
| BLCL | 0-A (70%), B-C-D (30%) |
| MELD | 9.4 |
| ***Biochemical data*** | |
| AST (U/l) | 41.3 |
| ALT (U/l) | 26 |
| γ-GT (U/l) | 57 |
| INR | 1.1 |
| Bilirubin (µmol/l) | 24 |
| AFP (ng/ml) | 16.3 |
| Albumin (g/l) | 38 |
| ***Oncological data*** | |
| Number of nodules | 1.6 |
| Dimension (mm) | 17.4 |

The values are expressed as median and inter-quartile range (IQR). For histological scores the range of variability is included.

BMI, body mass index; AST, alanine aminotransferase; ALT, aspartate aminotransferase; γ-GT, gamma-glutamyl transpeptidase.

**Table S4.** Oligonucleotide primers used for qPCR

| **Primer** | **Sense** | **Reverse** |
| --- | --- | --- |
| Human ***OSM*** | 5' TACTGCTCACACAGAGGACGC 3' | 5' CTATAGCCGCCATGCTCGC 3' |
| Human ***VEGF*** | 5' CCCACTGAGGAGTCCAACAT 3' | 5' TTTCTTGCGCTTTCGTTTTT 3' |
| Human ***HLA-G*** | 5' TTCTTCACATCCGTGTCCCG 3' | 5' TGACTGGGCCTTCACATTCC 3' |
| Human ***EPO*** | 5' GAGCCCAGAAGGAAGCCATC 3’ | 5' GCGGAAAGTGTCAGCAGTGA 3' |
| Human ***GAPDH*** | 5' TGGTATCGTGGAAGGACTCATGAC 3' | 5' ATGCCAGTGAGCTTCCCGTTCAGC 3' |
| Mouse ***Osm*** | 5' TTTCTCTGGGGATACCATCG 3' | 5' GGAGACACGATGGGCTATGT 3' |
| Mouse ***Osmr*** | 5' GGAGACACGATGGGCTATGT 3' | 5' CATCTGAGGTGATGGTGGTG 3' |
| Mouse ***Tbp*** | 5'CACATCACAGCTCCCCACCA 3' | 5'AGCGGAGAAGATGCTGGAAAC 3' |
| Mouse ***Cdh5*** | 5' ATTGAGACAGACCCCAAACG 3' | 5' TTCTGGTTTTCTGGCAGCTT 3' |
| Mouse ***Kdr*** | 5' GGCGGTGGTGACAGTATCTT 3' | 5' GTCACTGACAGAGGCGATGA 3' |
